# How preoperative upper gastrointestinal investigations affect the management of bariatric patients: results of a cohort study of 897 patients

**DOI:** 10.1007/s00464-024-11352-2

**Published:** 2024-10-28

**Authors:** Adisa Poljo, Jakob J. Reichl, Romano Schneider, Julian Süsstrunk, Jennifer M. Klasen, Lana Fourie, Adrian T. Billeter, Beat P. Müller, Ralph Peterli, Marko Kraljević

**Affiliations:** 1 Department of Visceral Surgery, Clarunis, University Digestive Health Care Center Basel, St. Clara Hospital and University Hospital Basel, Basel, Switzerland; 2 Department of General Internal Medicine, University Hospital Basel, Basel, Switzerland; 3 Department of Surgery, Lucerne Cantonal Hospital, Spitalstrasse, Lucerne, Switzerland

**Keywords:** Bariatric surgery, Obesity, Preoperative management, Gastric bypass, Gastric sleeve

## Abstract

**Introduction:**

Preoperative diagnostic protocols vary worldwide, some prioritizing safety while others question routine procedures. Building on prior research, this study explores the impact of diverse preoperative findings on bariatric management and procedure selection.

**Methods:**

In a retrospective analysis of prospective data of over 1000 bariatric surgery patients from January 2017 to December 2022 undergoing primary laparoscopic Roux-en-Y gastric bypass (LRYGB) or sleeve gastrectomy (LSG) were analyzed. Preoperative assessment included upper endoscopy, upper GI series, and esophageal manometry. Sonography data were excluded. The primary endpoint examined the influence of preoperative exams on procedure selection, the secondary endpoint evaluated their therapeutic impact.

**Results:**

897 patients (741 RYGB, 156 SG) were included. All underwent upper endoscopy, revealing common findings such as type C gastritis and reflux esophagitis. Upper endoscopy prompted a therapeutic consequence in 216 patients (24.3%), resulting in a number needed to screen (NNS) of 4.1. Upper GI series and manometry were more frequently performed before LSG. Upper GI series detected hiatal hernias and motility disorders but did not result in any change of procedures. Esophageal manometry found pathologies in 37 (25.3%) patients rising to 41.5% if symptoms were present. Overall, 16 (1.8%) patients experienced a change in the planned procedure, with 14 changes prompted by preoperative findings and two by technical difficulties.

**Conclusion:**

We advise routine upper endoscopies for all patients undergoing LRYGB or LSG, while reserving upper GI series only for selected cases. Manometry should be exclusively performed on symptomatic patients undergoing LSG, ensuring a balanced and individualized preoperative assessment.

**Graphical abstract:**

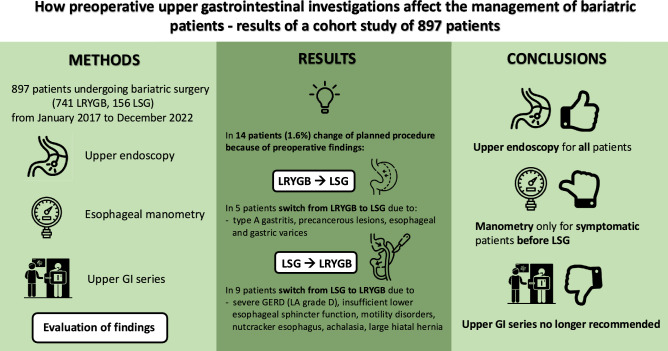

The prevalence of obesity has led to an increase in associated health risks, especially gastrointestinal pathologies like gallstones, hiatal hernias, gastritis, gastroesophageal reflux disease (GERD), peptic ulcer, Barrett’s esophagus (BE), and cancer raising concerns about potential abnormalities that could impact therapeutic approaches or pose technical challenges during bariatric surgery [1–3].

Preoperative diagnostic protocols for individuals undergoing bariatric surgery remain poorly defined and exhibit significant variations across countries and medical institutions. Some advocate for a comprehensive preoperative investigation, prioritizing patient safety over cost-effectiveness, while others question routine investigations due to the perceived clinical insignificance of their findings, healthcare costs, and concerns about efficiency [4, 5].

Efficient preoperative assessment aims to optimize procedure selection. The goal is to achieve effective weight loss and improve obesity-related conditions while reducing morbidity and mortality risks [6]. Several reports have already examined the range of various abnormalities in the upper GI tract among bariatric patients revealing that preoperative findings can have a substantial impact on the choice of bariatric procedures [7–10].

The discovery of inflammatory conditions, peptic ulcers or malignancies during examinations like esophagogastroduodenoscopy (EGD) may prompt a reevaluation of the selected bariatric procedure and introduce additional considerations. For instance, symptoms of GERD may exacerbate after sleeve gastrectomy (SG), marking severe GERD and large hiatal hernias as potential contraindications for SG [11]. The discovery of structural abnormalities through imaging studies introduces another layer of complexity, with anatomical anomalies potentially impacting the technical aspects of specific bariatric procedures.

Previously, we conducted a study on more than 1200 patients from 2007 to 2017 to assess the impact of preoperative investigations on managing bariatric patients [8]. Our findings indicated that routine preoperative upper GI endoscopy should be recommended for all patients. Additionally, we recommended conducting upper GI series and esophageal manometry solely for planned laparoscopic SG (LSG) cases, suggesting their exclusion for patients primarily scheduled for laparoscopic Roux-en-Y gastric bypass (LRYGB).

Building on these insights, this study applies previous findings to a new patient group, investigating the ongoing relevance of management recommendations. By analyzing endoscopic and radiologic examination results before bariatric surgery, the study aims to display pathologies and their influence on patient management and procedure selection.

## Materials and methods

The study received approval from the Ethics Committee of Northwestern Switzerland (reference number: 2018/00356). We entered data including demographic information, early morbidity records, and follow-up details on weight loss, co-morbidities, and complications on all bariatric patients who underwent surgery at our institution into a prospective database. Informed consent was obtained from all individuals participating in this study as a mandatory aspect of our hospital’s quality control procedures.

### Patients

In a retrospective examination of prospectively collected data, we analyzed all patients who underwent either LRYGB or LSG in our department from January 2017 to December 2022, comprising a total of over 1000 procedures. Patients undergoing gastric banding as their primary procedure or those undergoing revisional operations were excluded, leaving 897 patients for further analysis.

Following Swiss guidelines [12], we included individuals with a BMI ≥ 35 kg/m^2^ who did not respond to conservative treatment for 2 years, or 1 year in the case of a BMI ≥ 50 kg/m^2^.

Once patients meet the criteria for weight loss surgery, they begin an extensive preoperative assessment process. Our comprehensive approach to evaluating eligibility for bariatric and metabolic surgery involves a multidisciplinary and interprofessional team, encompassing endocrinologists, psychiatrists, registered nutritionists, and bariatric surgeons. The thorough preoperative assessment entails multiple appointments, educational sessions, additional laboratory and diagnostic testing, and targeted medical interventions tailored to the individual’s clinical needs.

### Preoperative examinations

In our department, we conduct preoperative examinations of the upper GI tract to assess anatomy and detect potential pathologies before surgery.

Before conducting any upper GI investigations, we engage in discussions with patients regarding their medical history and preferences to determine the most suitable surgical options. The preoperative investigations are adjusted to the chosen procedure. Nearly all patients undergo transabdominal sonography and upper endoscopy pre-surgery, with additional procedures like upper GI series for selected cases. In our current study protocol, we typically recommend GI series and manometry for all patients scheduled for LSG, based on findings from our previous research [8]. However, there are instances where our adherence to this protocol is not absolute. For instance, if upper endoscopy reveals no sign of a hiatal hernia, we sometimes refrain from conducting additional upper GI series. Moreover, the decision to perform additional evaluations is occasionally influenced by surgeon preferences.

Our standard practice aims to combine upper endoscopy and manometry in a single session. However, due to patient intolerance, manometry may be omitted in some cases. Furthermore, unforeseen changes in the planned procedure, such as switching from LRYGB to LSG due to preoperative findings (e.g., from endoscopy), lead to adjustments in our approach. In cases where patients are asymptomatic, we do not insist on performing manometry, as it would not impact management or outcomes.

LRYGB is recommended over LSG for severe reflux esophagitis, large hiatal hernias (> 4 cm), and motility disorders. SG is contraindicated for patients with BE, a precursor to esophageal adenocarcinoma, due to its potential to worsen GERD and progress BE [13, 14].

On the other hand, SG is suggested for patients with a very high BMI, requiring post-surgery upper endoscopy surveillance, experiencing micronutrient deficiency, diagnosed with Crohn’s disease, or having a history of extensive abdominal surgery.

Detection of HP during endoscopy is followed by eradication therapy prior to surgery. HP eradication is typically achieved with bismuth quadruple therapy, which consists of bismuth subcitrate, metronidazole, and tetracycline, in combination with a proton pump inhibitor (PPI). Eradication is confirmed four weeks later through stool polymerase chain reaction (PCR) testing.

We intentionally refrained from analyzing and assessing preoperative sonography, as this has been previously published [7, 8].

The primary endpoint focused on assessing how preoperative examinations influenced the choice of procedure. The secondary endpoint examined the impact of these examinations on the therapeutic consequences.

### Statistical analysis

Mean and standard deviation were used to summarize continuous data, while counts and percentages were used for categorical variables. Normality was examined using the Kolmogorov–Smirnov and Shapiro–Wilk tests. Characteristics between the groups were compared using Fisher exact test for categorical variables and Mann–Whitney U test for nonparametric variables. The impact of examination findings was presented as the number needed to screen (NNS), which is statistically defined as the number of people required to be screened to detect one adverse event.

All analyses were carried out using SPSS (Version 25, SPSS Inc., Chicago, IL, USA).

## Results

### Patient characteristics

Eight hundred and ninety-seven patients were included in this study, with 741 undergoing LRYGB and 156 LSG. Originally, LSG was planned in 169 and LRYGB in 728 patients. The mean age was similar between the groups, with 41.4 years for LRYGB and 43.3 years for LSG. Patients undergoing LRYGB were more often women with 73.0% (vs. 57.7% in LSG) and had a lower BMI (41.7 vs. 44.6 kg/m^2^) (Table 1).

**Table 1:** Demographic data

	LRYGB	LSG	Total	*p*
n (%)	741 (82.6%)	156 (17.4%)	897 (100.0%)	–
Age (years)	41.4 ± 12.4	43.3 ± 12.5	41.8 ± 12.4	0.09
Female sex (%)	541 (73.0%)	90 (57.7%)	631 (70.3%)	< 0.001**
Baseline BMI (kg/m^2^)	41.7 ± 5.7	44.6 ± 7.7	43.0 ± 6.2	0.02*

Values are expressed as means ± standard deviation

*LYRGB* laparoscopic Roux-en-Y gastric bypass; *LSG* laparoscopic sleeve gastrectomy; *BMI* body mass index

*p < 0.05; **p < 0.01

### Examination findings

#### Upper endoscopy

Upper endoscopy was performed in all patients and identified at least one pathological finding in 471 patients (63.6%) before LRYGB compared to 89 (57.1%) before LSG. The most common findings were type C gastritis (228; 30.8% for LRYGB vs. 89; 57.1% for LSG), reflux esophagitis (153; 20.9% vs. 22; 14.1%), and an HP infection (117; 15.8% vs. 28; 17.9%). The majority of patients (129; 73.7%) who presented with reflux esophagitis exhibited grade A in the Los Angeles (LA) classification. Among the patients undergoing LRYGB, 3 patients (2.0%) showed grade D compared to one patient (4.5%) undergoing LSG.

LRYGB was performed in the presence of BE in 13 (17.8%) patients and LSG in 3 patients (1.9%).

Less common findings included peptic ulcers, an intramural lipoma, intestinal metaplasia, cardia insufficiency, eosinophilic esophagitis, esophageal candidiasis, esophageal diverticulum, esophageal varices, ampullary adenoma, and celiac disease. The complete list is provided in Table 2.

**Table 2:** Findings in upper endoscopy

	LRYGB	LSG	Total	*p*
Examinations (n; %)	741; 100.0%	156; 100.0%	897; 100.0%	–
Normal examination (n; %)	270; 36.4%	67; 42.9%	337; 37.6%	0.07
Eosinophilic esophagitis (n; %)	3; 0.4%	1; 0.6%	4; 0.5%	0.54
Esophageal candidiasis (n; %)	1; 0.1%	0; 0.0%	1; 0.1%	1.00
Esophageal diverticulum (n; %)	1; 0.1%	0; 0.0%	1; 0.1%	1.00
Esophageal varices (n; %)	0; 0.0%	1; 0.6%	1; 0.1%	1.00
Cardia insufficiency (n; %)	9; 1.2%	3; 1.9%	12; 1.4%	0.45
Hiatal hernia (n; %)	80; 10.9%	69; 44.2%	149; 16.8%	< 0.001**
Barrett’s esophagus (n; %)	13; 17.8%	3; 1.9%	16; 1.8%	0.75
Reflux esophagitis (n; %)	153; 20.9%	22; 14.1%	175; 19.7%	
LA grade A	− 114; 74.5%	− 15; 68.2%	− 129; 73.7%	
LA grade B	− 31; 20.3%	− 6; 27.3%	− 37; 21.1%	
LA grade C	− 5; 3.3%	− 0; 0.0%	− 5; 2.9%	
LA grade D	− 3; 2.0	− 1; 4.5%	− 4; 2.3%	0.12
Helicobacter pylori infection (n; %)	117; 15.8%	28; 17.9%	145; 16.2%	0.31
Typ C gastritis (n; %)	228; 30.8%	89; 57.1%	317; 35.3%	< 0.001**
Type A gastritis (n; %)	1; 0.1%	2; 1.3%	3; 0.3%	0.08
Peptic ulcer (n; %)	4; 0.5%	2; 1.3%	6; 0.7	0.29
Intramural Lipoma (n; %)	1; 0.1%	0; 0.0%	1; 0.1%	0.82
Intestinal metaplasia (n; %)	1; 0.1%	3; 1.9%	4; 0.5%	0.02*
Portal hypertensive gastropathy (n; %)	1; 0.1%	0; 0.0%	1; 0.1%	0.82
Ampullary adenoma (n; %)	0; 0.0%	1; 0.6%	1; 0.1%	1.00
Celiac disease (n; %)	2; 0.3%	1; 0.6%	3; 0.3%	0.44
Changes in therapy due to upper endoscopy (yes; %)	176; 24.0%	40; 25.6%	216; 24.3%	0.54
HP-eradication	117; 66.5%	28; 70.0%	145; 67.1%	
Hiatal hernia repair	67; 38.1%	32; 80.0%	99; 45.8%	
Repositioning of stomach	1; 0.1%	0; 0.0%	1; 0.5%	
Postoperative diverticulectomy	1; 0.1%	0; 0.0%	1; 0.5%	
LSG instead of planned LRYGB	–	5; 12.5%	5; 2.3%	
LRYGB instead of planned LSG	5; 2.8%	–	5; 2.3%	
NNS	4.2	3.9	4.1	0.54

*LYRGB* laparoscopic Roux-en-Y gastric bypass; *LSG* laparoscopic sleeve gastrectomy; *GERD* gastroesophageal reflux disease; *NNS* number needed to screen (for change in therapy)

^*^p < 0.05; **p < 0.01

In total, upper endoscopy prompted a therapeutic change in 216 patients (24.3%), yielding a number needed to screen (NNS) of 4.1, with no significant differences between the two groups. Among these, 145 patients (67.1%) predominantly underwent HP eradication therapy, followed by hiatal hernia repair. In 5 patients, there was a switch from planned LRYGB to LSG, and from planned LSG to LRYGB, respectively.

#### Upper GI series

Upper GI series were more frequently conducted before planned LSG (82; 11.1% before RYGB vs. 106; 67.9%). Pathologic findings were observed in 36 patients (43.9%) before LRYGB and 38 patients (35.8%) before LSG. The most common findings were hiatal hernias (32; 39.0% vs. 34; 32.1%) and motility disorders (4; 4.9% vs. 7; 6.6%). Therapeutic consequences involved hernia repair and repositioning of an upside-down stomach, resulting in an overall NNS of 3.6. In none of the cases did the findings in the upper GI series result in a change of surgical procedure. Further details are provided in Table 3.

**Table 3:** Findings in upper GI series

	LRYGB	LSG	Total	*p*
Examinations (n; %)	82; 11.1%	106; 67.9%	188; 21.0%	–
Normal examinations (n; %)	46; 56.1%	68; 64.2%	114; 60.6%	0.29
Hiatal hernia (n; %)	32; 39.0%	34; 32.1%	66; 35.1%	0.36
Motility disorders (n; %)	4; 4.9%	7; 6.6%	11; 5.9%	0.76
Juxtapapillary duodenal diverticula (n; %)	1; 1.2%	0; 0.0%	1; 0.5%	0.44
Upside-down stomach (n; %)	1; 1.2%	0; 0.0%	1; 0.5%	0.44
Esophageal diverticulum (n; %)	1; 1.2%	0; 0.0%	1; 0.5%	0.44
Changes in therapy due to upper GI series (yes; %)	25; 30.5%	27; 25.5%	52; 27.7%	0.51
Hiatal hernia repair	24; 96.0%	27; 100.0%	51; 98.1%	
Repositioning of stomach	1; 0.0%	0; 0.0%	1; 1.9%	
LSG instead of planned LRYGB	0; 0.0%	0; 0.0%	0; 0.0%	
LRYGB instead of planned LSG	0; 0.0%	0; 0.0%	0; 0.0%	
NNS	3.3	3.9	3.6	0.51

*LYRGB* laparoscopic Roux-en-Y gastric bypass; *LSG* laparoscopic sleeve gastrectomy; *NNS* number needed to screen (for change in therapy)

#### Esophageal manometry

Esophageal manometry was primarily performed when LSG was scheduled. Originally, 13 more patients were initially planned for LSG preoperatively, but ultimately underwent LRYGB due to pathological findings in manometry (*n* = 6) and a change in their preference (*n* = 7).

Overall, 132 (78.1%) patients underwent preoperative manometry before planned LSG compared to only 13 (1.8%) patients before LRYGB.

Among the patients initially scheduled for LSG, 30 reported symptoms indicative of reflux, regurgitation, or dysphagia. Manometry revealed a pathology in 14 of them (46.6%). In 5 patients out of these 14 (35.7%), this pathology prompted a shift from planned LSG to LRYGB (NNS = 6 in symptomatic patients).

Focusing on patients who finally underwent LSG, manometry showed pathological findings in 27 (22.4%) compared to 10 (37.0%) in LRYGB. Findings included a hypertensive or insufficient lower esophageal sphincter (LES), motility disorders, achalasia, and nutcracker esophagus. Due to these findings, 7 (4.8%) patients who were initially planned for LSG had to undergo LRYGB (NNS = 22). Reasons for a change of procedure were an insufficient LES in 3 patients, motility disorders in 2 patients and achalasia and nutcracker esophagus in one, respectively. Out of these 7 patients, 5 had symptoms.

Interestingly, only in 2 out of 105 (1.9%) asymptomatic patients, a clinically relevant motility disorder was identified. Notably, one of them had additional erosive reflux esophagitis, which also played a crucial role in the decision to change the surgical procedure. Ultimately, in only 1 out of 105 (1.0%) asymptomatic patients, the manometry alone was decisive for the change of procedure. The results are presented in Table 4.

**Table 4:** Findings in esophageal manometry examination

	LRYGB	LSG	Total	p
Examinations (n; %)	27; 3.6%	119; 76.3%	146; 16.3%	0.003
Initially planned for LRYGB/LSG	13; 1.8%	132; 78.1%	–	
Normal examinations^a^ (n; %)	17; 63.0%	92; 77.3%	109; 74.7%	0.19
Symptoms present^a^ (n; %)	16; 59.3%	25; 21.0%	41; 28.1%	< 0.001**
Pathologic examination, if symptoms present (n; %)	8; 50.0%	9; 36.0%	17; 41.5%	
No symptoms present^a^ (n; %)	11; 40.7%	94; 79.0%	105; 71.9%	< 0.001**
Pathologic examination, if no symptoms present (n; %)	2; 18.2%	18; 19.1%	20; 19.0%	
Hypertensive esophageal spincter^a^ (n; %)	0: 0.0%	2; 1.7%	2; 1.4%	1.00
Insufficient esophageal spincter^a^ (n; %)	6; 22.2%	13; 10.9%	19; 13.0%	0.20
Motility disorder^a^ (n; %)	1; 3.7%	12; 10.1%	13; 8.9%	0.49
Achalasia^a^ (n; %)	2; 7.4%	0; 0.0%	2; 1.4%	0.03*
Nutcracker esophagus^a^ (n; %)	1; 3.7%	0; 0.0%	1; 0.7%	0.41
Changes in therapy due to manometry^a^ (yes; %)	0, 0.0%	7; 5.3%	7; 4.8%	
LSG instead of planned LRYGB	0; 0.0%	0; 0.0%	0; 0.0%	
LRYGB instead of planned LSG if symptomatic	0; 0.0%	7; 100.0%	7; 100.0%	-
NNS^a^ -In symptomatic patients	–	18.9 6.0		-

*LYRGB* laparoscopic Roux-en-Y gastric bypass; *LSG* laparoscopic sleeve gastrectomy; *NNS* number needed to screen (for change in therapy)

^*^p < 0.05; **p < 0.01

^a^The findings apply to patients in whom the respective surgery was actually performed

#### Change of surgical procedure

In total, the originally planned procedure had to be changed in 16 patients (1.8%). Out of these, 14 changes were caused by findings in the preoperative examinations and two by technical intraoperative difficulties.

In 9 patients for whom LRYGB was initially planned, the switch to LSG was necessary due to findings of type A gastritis, technical difficulties, precancerous lesions (intestinal metaplasia and ampullary adenoma), and esophageal and gastric varices (Table 5).

**Table 5:** LSG instead of planned LRYGB

Total (n; %)	7; 4.5%
Type A Gastritis (n; %)	2; 1.3%
Technical difficulties (n; %)	2; 1.3%
Precancerous lesions (n; %)	2; 1.3%
Esophageal and gastric varices (n; %)	1; 0.1%

*LYRGB* laparoscopic Roux-en-Y gastric bypass; *LSG* laparoscopic sleeve gastrectomy;

In 9 patients for whom LSG was originally planned, the decision to opt for LRYGB was made due to severe reflux esophagitis (LA grade D), an insufficient lower esophageal sphincter, motility disorders, nutcracker esophagus, achalasia, and a large hiatal hernia (Table 6). In one patient there were two reasons for change in procedure (insufficient esophageal sphincter and motility disorder).

**Table 6:** LRYGB instead of LSG

Total (n; %)	9; 1.0%
Severe reflux esophagitis (n; %)	3; 0.4%
Insufficient esophageal sphincter (n; %)	3; 0.4%
Motility disorder (n; %)	1; 0.2%
Nutcracker esophagus (n; %)	1; 0.1%
Achalasia (n; %)	1; 0.1%
Large hiatal hernia (n; %)	1; 0.1%

*LYRGB* laparoscopic Roux-en-Y gastric bypass; *LSG* laparoscopic sleeve gastrectomy; *GERD* gastroesophageal reflux disease

## Discussion

Our study aimed to implement the management recommendations derived from our previous research on preoperative investigations [8]. The findings revealed notable pathological results, prompting various modifications in the therapeutic approach including adjusting medication, repairing hiatal hernias, performing diverticulectomy and finally changing the initially planned surgical procedure.

Multiple studies have already investigated various abnormalities in the upper GI tract among bariatric patients [7–10]. However, despite ongoing research, there is still no consensus or standardized approach to preoperative examinations before bariatric surgery [15].

Moulla et al. examined preoperative EGD in 636 patients, revealing a change in the operative strategy in 1.6% with detection of esophageal adenocarcinomas in three cases (0.5%) [6]. Other studies emphasize the importance of detecting prevalent BE. In a study of 169 patients with a median 7.0 ± 1.5 years follow-up, the LSG group (*n* = 83) had 3 cases of de novo BE, while the LRYGB group (*n* = 86) had 1 case (3.6% versus 1.2%). Additionally, the LSG group reported higher prevalence of reflux symptoms and moderate-to-severe reflux esophagitis despite greater proton pump inhibitor use [16].

In our study, precancerous lesions were identified in 5 patients (0.6%), including 4 intestinal metaplasias and 1 ampullary adenoma. BE was observed in 16 patients (1.8%), with no detection of dysplasia requiring further treatment. Among these, BE was identified in three patients who underwent LSG. Initially, LRYGB had been recommended for these patients preoperatively. However, they chose LSG based on personal preference and positive experiences shared by family members or friends who had successful outcomes with LSG. All patients were informed about their increased risk of dysplasia and the potential development of Barrett’s carcinoma. Furthermore, it was advised that these patients undergo regular endoscopic surveillance to monitor for any progression of BE or the development of dysplasia.

According to the Bariatric Outcomes Longitudinal Database, LRYGB is more effective than other weight loss procedures in reducing GERD symptoms [14]. 5-year outcomes of combined data from two randomized clinical trials (SLEEVEPASS and SM-BOSS) revealed that around 8% of patients undergoing SG necessitated conversion to RYGB due to GERD [17].

In our study, we simplified the categorization of reflux esophagitis findings by summing all LA grades up as "reflux esophagitis" for analysis. However, according to the latest Lyon consensus, only LA grades C and D offer evidence of reflux [18]. Applying this refined criterion, we found that only 8 out of 153 LRYGB patients and 1 out of 22 SG patients should have been diagnosed with reflux esophagitis, significantly reducing reflux esophagitis prevalence from 5.2 to 0.9% in RYGB and 14.1% to 4.5% in SG. Despite having LA grade D reflux esophagitis, one patient chose LSG against our recommendation. Surprisingly, a follow-up endoscopy two years later showed grade A reflux esophagitis, suggesting an improvement in GERD-related symptoms. This supports data on the role of LSG in improving GERD symptoms [19, 20].

Our study found a 16.8% prevalence of hiatal hernias detected via EGD and 35.1% via upper GI series. This discrepancy may be attributed to the lower diagnostic accuracy of upper GI series for hiatal hernias [21]. However, our results are consistent with rates reported in other studies ranging between 20 and 40% [21, 22]. Hiatal hernias were more prevalent in patients undergoing LSG compared to those undergoing RYGB with 10.9% and 44.2% (diagnosed via EGD), respectively. Research suggests that hiatal hernias smaller than 2 cm may not be clinically significant [23]. However, as we lacked information on hernia size, we cannot determine the clinical significance of the detected hernias in our study. Intraoperatively, a higher proportion of hiatal hernias diagnosed before RYGB were fixed during surgery (67 out of 80; 83.8%) compared to those diagnosed before SG (32 out of 69; 46.4%), indicating potential overdiagnosis of hiatal hernias before SG. It should be noted that many, if not all, hiatal hernias would likely be identified intraoperatively, which may suggest that preoperative endoscopy and upper GI series do not contribute significantly to a change in therapy for these patients. This observation might imply that a larger number of patients would need to be screened to achieve a meaningful change in therapy. However, the primary strength of preoperative upper endoscopy lies not in the detection of hiatal hernias, but in its ability to diagnose dysplasias, malignancies, and severe reflux esophagitis, which we consider essential before performing bariatric surgery. In our previous study, for example, we identified one case of Barrett’s high-grade dysplasia, two cases of Barrett’s carcinoma, and one case of stomach cancer in asymptomatic patients [8]. In this regard, it is also important to note the significant variation in the costs of upper GI endoscopy, ranging from $3,000 to $6,000 in the United States, compared to approximately 350 CHF at our hospital, which may account for differences in the utilization of upper endoscopy across institutions. The therapeutic benefit of an upper GI series is therefore questionable, given that most relevant hiatal hernias are identified during the upper endoscopy and operative procedure itself. Overall, hiatal hernias were discovered intraoperatively in 21 patients (2.3%) who had not been identified in preoperative investigations. Nevertheless, knowing about a large hiatal hernia preoperatively still can be helpful for assembling the OR team and estimating the duration of the procedure.

The utilization of upper GI series and esophageal manometry is subject to debate. In our series, no alterations to the planned surgical procedure were made based on findings in the upper GI series.

During long-term follow-up, the preoperative manometric data of the esophageal body can be predictive of the development of postoperative esophageal dilation, stasis and aggravation or new onset of symptoms [24]. Patients with asymptomatic, compensated GERD but low-pressure LES are at high risk to develop GERD symptoms postoperatively which has to be taken into account when planning LSG [25].

Our data revealed that screening patients with esophageal manometry, whether symptomatic or not, resulted in pathological findings in 37 patients (25.3%), leading to a procedural change in 7 (4.8%) patients. When symptoms were present, the detection rate increased to 41.5%. These findings align with other studies suggesting that performing manometry before bariatric surgery in patients with esophageal symptoms, such as heartburn, regurgitation, dysphagia, and non-cardiac chest pain, may indicate abnormal esophageal motility [26].

It has been suggested that LRYGB may be the preferred procedure for patients with motility disorders, as reduced LES pressure following LSG could exacerbate GERD, thereby complicating esophageal motility disorders [27–29]. Achalasia has already been linked to unfavorable outcomes following bariatric surgery, and its management prior to surgery appears to ameliorate the postoperative course [29]. In this context, it is important to consider that the progression of achalasia could require Heller myotomy with Dor fundoplication, which is not feasible after LSG [30]. Our findings indicate that maintaining the practice of routine manometry before LSG mainly yields advantages for symptomatic patients. However, it seems reasonable to extend the necessity of manometry in any patient with symptoms suggestive of severe motility disorder before any bariatric procedure [26]. While manometry did not reveal significant value in asymptomatic patients scheduled for LSG or LRYGB, neither upper GI series nor manometry influenced surgical decisions for those undergoing LRYGB.

Prioritizing cost-effectiveness as a rationale for deciding upon specific preoperative examinations is recommended by some authors [5, 31]. While cost considerations are undoubtedly important, it is crucial to acknowledge that certain findings can profoundly impact a patient’s life, even if they occur rarely. In our case, findings, such as precancerous lesions, can have a significant and lasting effect, particularly if they are located in the gastric remnant. The challenging endoscopical access associated with LRYGB could potentially carry the risk of progression to carcinoma. Furthermore, the timely diagnosis of severe GERD, BE, and severe motility disorders before LSG can prevent patients from requiring a later conversion to LRYGB [32, 33].

Building upon the findings of our previous study [8], which indicated a lack of clinical relevance in performing upper GI series and manometry, particularly when LRYGB is planned, the current study provides further evidence supporting the safety of foregoing upper GI series in all patients. Hence, we suggest to conduct manometry exclusively in symptomatic patients, aligning with the existing ASMBS guidelines that already advocate a similar approach for upper GI endoscopy [15]. The lack of a universal consensus highlights the necessity for additional research and development in this field to establish more standardized and evidence-based protocols for the preoperative assessment in bariatric surgery.

### Limitations

Our study’s limitations stem primarily from its retrospective nature, which poses constraints on data collection and introduces potential biases. The generalizability of our findings is limited due to the lack of international consensus on standardized preoperative investigations for bariatric surgery, compounded by variations arising from individual surgeon preferences and healthcare systems. Additionally, differences in healthcare costs and regional disparities further complicate the approach to preoperative assessments. Diagnostic findings may be operator-dependent and vary due to personal interpretations, and differences between clinics. Moreover, the definition of clinically significant findings lacks clarity and relies on subjective surgeon interpretation.

## Conclusion

In conclusion, we recommend the standard implementation of upper endoscopy in the preoperative evaluation of all patients undergoing LRYGB or LSG. We discourage the routine use of upper GI series and suggest its selective application based on specific clinical indications. Furthermore, we propose the integration of manometry only in symptomatic patients prior to undergoing LSG. This tailored approach ensures a reasonable use of resources while maintaining a thorough and individualized preoperative assessment for optimal patient outcomes.

## Data Availability

The data and codes relating to the study are available from the corresponding author upon reasonable request.
